# Eye Injuries

**Published:** 1910-04-09

**Authors:** Philip A. Harry


### EYE INJURIES.

By PHILIP A. HARRY, M.D., D.P.H.

This is a class of injury demanding special attention, not only from the importance of the organ injured, but also from the points of view of prognosis and of the Workmen's Compensation Acts. In manufacturing districts 20 per cent. or more of eye cases are due to trauma. During 1909 nearly 200 cases of serious injury to the eye were admitted, * forming 17.5 per cent. of the total eye in-patients; this does not include burns nor the results of corneal foreign bodies or abrasions.

A survey of these cases shows that they may be most conveniently classified into contusions, perforating or punctured wounds, penetrating wounds with retained foreign body, and ruptures. Contusions form a large proportion of eye injuries; septic infection from without does not take place. The anterior, or antero-temporal, part of the globe is the usual seat of impact. Blows on the temporal aspect more often than not come under the head of ruptures, and will be mentioned later.

* The Leeds General Infirmary

A sudden blow on the front of the eye, usually with an object as large as, or larger than the globe, causes a flattening of the cornea and a momentary rise of tension. The anterior chamber is reduced in size and the aqueous is forced backwards against either the lens or the iris. According to the severity of the blow there will be produced a dislocation backwards of the lens, a stretching of the constrictor muscle of the iris, a guttering or a tucking backwards of the iris into the posterior chamber, or an actual tearing of the iris from its attachment to the ciliary body (iridodialysis). In nearly every case there is an escape of blood into the anterior chamber from the vessels of the iris. This hæmorrhage is usually slight, and in 24 or 36 hours it collects in the lowest part of the anterior chamber (hyphæma).

The sudden stretching of the sphincter muscle of the pupil causes mydriasis; it is very rare for the dilatation to be regular, and its disappearance is very gradual. In these cases a rupture of the constrictor muscle may often be made out with the Wurdeman transilluminator. General injection of the eyeball is the result of the reaction to the injury rather than of the momentary rise and fall of tension which causes the hæmorrhage from the iris. When there has been separation of the iris from the ciliary body the bleeding is very marked, the anterior and sometimes the posterior chamber being filled with blood. Subconjunctival ecchymosis is only seen when there has been injury to the orbit or orbital tissues. Iritis is not usual except when the eye has been previously diseased or in the presence of marked constitutional disease.

Eserine, gr. j. ad ℥ j. and dionin 4 per cent., either separate or combined, is the best treatment. The eserine renews the activity of the constrictor pupillæ, while the dionin hastens the disappearance of the hæmorrhage.

Dislocation of the lens is diagnosed by the tremulous iris, produced in great part by the loss of support of the lens. The lens may be partially or totally dislocated; in the latter case it gradually falls to the lower part of the vitreous. In a few cases the lens is dislocated forward, and slips through the widely stretched pupil into the anterior chamber. Such a dislocated lens gradually becomes opaque. When there has been rupture of the anterior capsule opacity is rapidly produced; occasionally a rent can be seen in this position, in other cases it is possible that the tearing away of the suspensory fibres from the lens surface is the starting point of the opacities.

Should the blow be received on the closed lids, they will be discoloured; if received directly on the cornea a corneal abrasion will be noticed on staining with fluorescin. In several cases a peculiar star-shaped opacity has been seen, possibly due to cracks in Descemet's membrane.

The next stage to this where there is an actual solution of continuity of the cornea brings the case under the class of ruptures.

Fundus changes are not uncommon; the so-called commotio retinæ, ruptures of the choroid concentric with the disc, and circular or oval hæmorrhages in the macular region are the most frequent. There is usually some cloudiness of the vitreous produced by hæmorrhage or exudate. Detachment of the retina, on the other hand, is extremely rare, except in cases of abnormal or fluid vitreous or in unsound eyes.

The objects producing this class of injury are usually as large as, or larger than, the eye. They include a great variety of things—projecting parts of furniture, fists, horns, large pieces of metal, stones, handles of tools, etc.

Perforating or punctured wounds are produced by sharp objects, nearly always bigger than the eyeball, such as penknives, hatpins, forks, pieces of glass of different kinds, etc. In the periphery of the cornea these wounds are practically always complicated by prolapse of the iris produced by the sudden escape of aqueous. Hyphæma is present from injury to the iris. In the majority of the cases in this class the lens is wounded and a traumatic cataract is produced. Wounds in or about the centre of the cornea are complicated only by injury to the lens. The prognosis is very good when only these three—cornea, iris, and lens—are injured; when, however, the site of puncture or perforation is the sclera, or in the few cases when the object penetrates the lens as well as cornea and iris, the prognosis becomes grave because infection of the vitreous is more than likely. This tissue is eminently suitable to the growth of organisms, and its infection always ends in destruction of the eye sooner or later, if not by the active spread to the coats of the eyeball and to the orbital tissues causing panophthalmitis, then by reduction of the normal tension, ending eventually in a collapsed and shrunken globe.

If seen at the time of injury or soon after the conjunctival sac should be carefully douched out with a weak antiseptic lotion. Iris prolapsed beyond the surface of the cornea should be pulled slightly outwards and snipped off with suitable instruments. Should the iris not bulge beyond the surface of the cornea, eserine gr. ij. and cocaine grs. x. ad ℥j may be tried, provided the case be seen within twenty-four hours; after twenty-four hours, and in all other cases, atropine ½ or 1 per cent. should be instilled. Cold pads continuously, or at intervals, are useful for the first day or two after the accident. Two pieces of lint two or three inches square, stitched on three sides and containing several small pieces of ice, form a very satisfactory cold pad.

Sharp objects smaller than the eye coming with sufficient velocity to penetrate its coats usually remain in the globe. In this class of injury it is always advisable to treat the case as though the eye contained a foreign body until confirmed by radiography or transillumination. These penetrating foreign bodies are fortunately nearly always bits of steel; in a few cases the retained particles are of brass, lead, and glass. The smallest piece was a chipping of steel one millimetre long and half a millimetre broad.

In all penetrating wounds and perforating wounds with infection of the vitreous, iritis is soon set up, followed by cyclitis. A tender eyeball with ciliary congestion, pus in the anterior chamber, and other symptoms of iridocyclitis are sure signs of a retained foreign body. When the foreign body is of steel or iron the magnet must be used, whether the diagnosis be confirmed or not by radiography, and as soon as possible after the accident. The smaller magnets have been found more useful than the giant magnet, especially for the more minute foreign bodies. The point should be entered through the wound if there is one visible or through a wound made for the purpose in the sclera. After the extraction of the foreign body, treatment should be continued for the iridocyclitis and vitreous infection with dionin, subconjunctival injections of mercuric cyanide, Bier's suction hyperæmia, mercury internally, etc.

Eyes showing symptoms of iridocyclitis, from which the magnet fails to extract the foreign body, should be removed to prevent the risk of sympathetic disease. One case of sympathetic iridocyclitis was admitted during 1909. Even an eye possessing fair vision should be sacrificed if several attempts with large and small magnets have been unsuccessful in extracting a foreign body which has been seen by transillumination and repeated radiography. This applies to cases where the uninjured eye possesses good vision.

Ruptures of the eyeball are chiefly produced by large objects usually larger than the eye. In one case a piece of steel smaller than the eye ruptured the globe and buried itself in the upper part of the orbit. It was a chipping from the hammer of the workman next to the patient. He was unfortunate enough to sustain a perforating wound in the remaining eye soon after his return to work. A blow on the front of the eye may cause a break in the cornea by direct violence a stage further than the cracks mentioned above under contusions. A blow received on the outer side of the eyeball usually causes the eye to burst on the inner side, possibly by impact of the globe against the orbital margin. The rupture takes place in the sclera near to its junction with the cornea.

These injuries are usually very severe; if the tear is small and there is no escape of the contents of the globe it should be covered with a flap of conjunctiva kept in place by fine silk sutures. However, there is very often an escape of vitreous; occasionally the lens and more rarely the iris and ciliary region become prolapsed. The iris may be partially or totally torn from its attachment. In the latter case the eye is so destroyed that it is hopeless to attempt to save it. Moreover, the patient will be able to return to work all the sooner, and the worry and anxiety for all parties concerned is lessened when excision is performed.

